# Spinal Metastasis Pain Surveillance: A Comprehensive Imaging-Based Tool Design for Evaluating Metastatic Burden and Guiding Therapeutic Strategies

**DOI:** 10.7150/ijms.103916

**Published:** 2025-01-13

**Authors:** Shuxin Kong, Aishi Deng, Zeyin Guo, Lijia Ma, Xi Su, Junwei Cui, Yongkang Ou, Jinghua Liu, Tao Qin, Zeng Fang

**Affiliations:** 1 Department of Breast Surgery, Zhengzhou University People's Hospital (Henan Provincial People's Hospital), Zhengzhou, China.; 2 Department of Breast and Thyroid Surgery, Peking University ShenZhen Hospital, China.; 3 Tongxinling Community Health Service Center, Futian District, The 8 th affiliated hospital of Sun yet-sen University, China.; 4 Department of Imaging, Zhengzhou University People's Hospital (Henan Provincial People's Hospital), Zhengzhou, Zhengzhou, China.; 5 Guang'anmen Hospital, China Academy of Chinese Medical Sciences, Beijing, 100053, China.; 6 Department of Hepato-Biliary-Pancreatic Surgery, Henan Provincial People's Hospital (People's Hospital of Zhengzhou University), Zhengzhou, China.

**Keywords:** Spine metastases, Bone pain, MRI

## Abstract

**Background:** The current research aims to elucidate the interplay between the anatomical distribution of spinal metastases, MRI features, and the intensity of bone pain in patients with breast cancer.

**Methods:** A retrospective analysis was used on a cohort of 45 breast cancer patients with verified spinal metastases, examining the relationship between metastatic locations, MRI-derived metrics, and bone pain scores. The Visual Analogue Scale (VAS) was conducted to measure the severity of bone pain.

**Results:** The results revealed a significant association between lumbar spine metastases and elevated pain scores, outpacing those observed in thoracic and cervical regions. Furthermore, a strong correlation was found between the multiplicity of metastatic sites and the ratio of high-intensity areas on MRI, both of which were predictive of increased pain severity.

**Conclusions:** The study's outcomes indicate that distinct MRI profiles, including the number and location of spinal metastases, can serve as prognostic indicators of bone pain intensity in breast cancer patients. Our data highlighted the need for personalized pain management strategies and targeted interventions tailored to specific imaging characteristics. Ultimately, this research underscores the dual role of MRI in both detecting spinal metastases and informing symptom management, with the potential to augment the overall well-being of breast cancer patients with spinal involvement.

## Introduction

Breast cancer is a complex and multifaceted disease that can have far-reaching consequences for patients, including the development of bone metastases 1. The skeletal system is a common site for metastatic spread, with approximately 70% of patients experiencing bone involvement at some point during their illness 2, 3. The spine, in particular, is a frequent location for metastases 4-6. However, migration can also happen in the cervical and lumbar spine, each with its own unique set of challenges and implications for patient care.

The presence of spinal metastases might make a profound influence on a patient, causing a range of symptoms including local pain, spinal instability, and neurological impairment. In some cases, spinal metastases can lead to neurologically compromising spinal lesions 7. This can cause significant pain, neurological dysfunction, and even paralysis, all of which will make a devastating influence on a personal lifestyle and habits and overall well-being 8, 9.

Despite the availability of treatments such as radiological treatments and orthopedic procedures, the management of symptomatic spinal cord compression remains a significant challenge 10, 11. The development of spinal metastases can require unscheduled treatments, which can disrupt the treatment schedule for the primary cancer and impede the patient's overall care. As a result, there is a growing recognition of the importance of bone management through a comprehensive strategy to mitigate bone-related complications and to improve patient outcomes 12.

A key aspect of bone management is the early diagnosis of symptomatic changes and the effective management of related pain or system therapy. However, there is still much to be learned about the factors that contribute to spinal metastasis and the relationship between the location of metastases, imaging characteristics, and the severity of associated bone pain 12, 13. For example, research has shown that individuals with malignant spinal canal encroachment are more likely to experience symptoms in the thoracic spine, although a significant minority of cases occur in the cervical spine, potentially leading to greater morbidity and mortality 14.

Employment of cutting-edge diagnostic modalities like high-field nuclear magnetic resonance has revolutionized the diagnosis and characterization of spinal metastases 15. MRI provides superior soft tissue contrast and detailed bone marrow imaging, making it an essential tool in the investigation of suspected cases of metastatic spinal cord compression. However, there is still a need for further research into the potential roles of specific MRI features in the assessment of bone pain caused by spinal cord compression.

This research seeks to bridge the existing information deficit by exploring the relationship between specific MRI features, such as the ratio of high-intensity areas in spinal metastases, and the intensity of bone pain. Additionally, the study will investigate the anatomical site of the metastasis within the spinal column and its potential impact on pain severity 16. By elucidating the complex interplay between imaging characteristics of spinal metastasis and the clinical manifestation of bone pain, this research endeavors to inform the creation of enhanced analgesic protocols and improve patient care and quality of life 17.

Ultimately, the goal of this Investigation is to offer clinicians with a more nuanced understanding of the factors that contribute to spinal metastasis and the symptoms that patients experience. By informing clinicians about the potential severity of pain based on MRI findings, this study aims to aid in anticipatory pain management strategies and improve patient outcomes. Furthermore, the study seeks to provide insights into the pathophysiological mechanisms of pain generation in spinal metastasis, guiding future therapeutic interventions and contributing to the development of more effective treatments for metastatic breast cancer.

## Materials and Methods

### Patients

To ensure the integrity and confidentiality of patient information, a comprehensive data retrieval process was implemented, involving the collection of medical records from patients who had undergone surgical procedures for breast cancer at these two hospitals. As an added precaution, all patient records were thoroughly anonymized and deidentified prior to analysis, thereby safeguarding patient privacy and confidentiality. In light of the study's archival nature, the Human Subjects Committee dispensed with the necessity for formal patient assent, as the research was conducted using existing medical records and did not involve any direct patient interaction or intervention 18.

A rigorous patient selection process was undertaken to identify a cohort of patients who met the specified inclusion criteria. Specifically, the study focused on patients who had been diagnosed with breast cancer and had subsequently developed spine metastases, as confirmed by clinical diagnosis and histological examination 19. To be eligible for inclusion, patients were required to have undergone MRI examinations at either Peking University Shenzhen Hospital or Henan Provincial People's Hospital between November 2014 and October 2023. A total of 45 patients met the inclusion criteria, which included: (1) a confirmed primary breast cancer diagnosis, as verified through histological examination; (2) a clinical diagnosis of spine metastases, as evidenced by relevant medical records and imaging data; and (3) the availability of comprehensive clinicopathologic features and image data for MRI examinations, which were meticulously recorded and stored in the patient database.

To guarantee the validity and trustworthiness of the research outcomes, a set of exclusion criteria was established to identify patients who did not meet the specified requirements. Patients were excluded from the study if they had been diagnosed with another type of malignancy, as this could potentially confound the results and introduce unnecessary variability 20. Additionally, patients with unclear or uncertain diagnoses of spine metastases were excluded, as were those without access to electronic image files of their MRI examinations, which were essential for conducting a thorough analysis of the data 21. By applying these strict inclusion and exclusion criteria, the study aimed to create a well-defined and homogeneous patient cohort, thereby enhancing the validity and generalizability of the research findings.

### Image analysis protocol

A thorough examination of the images was conducted by two seasoned medical radiologists, who utilized an advanced Xeleris workstation (GE Healthcare) to meticulously review the scans. The radiologists carefully documented the locations and quantities of spine metastases, as reported in the medical records 22. To quantify the extent of high-intensity areas within the spinal cord, the radiologists employed ImageJ software to calculate the proportion of high-intensity areas and the entire cord in the T2 scan images. This calculation enabled the determination of the Ratio of High Intensity Area (RHI), which was computed as the proportion of high-intensity area divided by the proportion of the whole cord 23. This metric provided a standardized measure of the high-intensity area within the spinal cord, allowing for a more nuanced understanding of the metastatic lesions.

### Pain assessment methodology

A comprehensive review of the medical records was conducted to determine the pain assessments for each patient. The Visual Analogue Scale (VAS) was used to evaluate the pain intensity associated with each lesion, with scores ranging from 0 (indicating no pain) to 10 (representing maximum pain) 24. A pair of medical specialists, including a junior doctor and an experienced clinician, independently assessed the pain intensity and recorded their findings in the medical records. Notably, both physicians were blinded to the changes in the MRI examination, ensuring that their assessments were unbiased and based solely on the clinical presentation of the patient. The initial pain intensity was subsequently categorized into four distinct levels: none (0), mild (1-3), moderate (4-7), or severe (8-10) 25. This categorization facilitated a more detailed analysis of the relationship between pain intensity and clinical features of the spine metastases.

### Statistical analysis approach

To investigate the relationships between pain scores and clinical features of the spine metastases, the researchers employed Pearson's correlation coefficient 26. This statistical method enabled the identification of correlations between the pain intensity and various clinical parameters, such as the distribution and quantity of tumor spread, along with the high-intensity area ratio 27, 28. Additionally, one-way analysis of variance was used to examine the relationships among the metastasis location, numbers, RHI, and severity of pain. Data analysis was conducted utilizing a specialized statistical package, specifically version 19.0 of a prominent software platform developed by a leading analytics firm based in Chicago, which provided a robust and reliable platform for data analysis. The researchers also conducted other analyses, including descriptive statistics and data visualization, to provide a comprehensive understanding of the data and to identify potential trends and patterns 29.

## Results

### Clinical characteristics

A cohort of 45 patients with diagnosed breast cancer and radiologically confirmed spinal metastatic disease were included in this study, all of whom were female, with a median age of 51 years (Table 1). The median time from breast cancer diagnosis to the development of spinal metastases was 27 months. The most common histologic subtype was Luminal B (69%), followed by TNBC (13%), Luminal A (9%), and Her-2 positive (9%). Among these patients, 87% experienced bone pain, and 90% developed pain in a metachronous pattern following spinal metastasis diagnosis. Regarding the distribution of metastases, 58% of patients had lesions in multiple spinal regions, whereas the lumbar spine was the most frequently affected single region (16%). Systemic therapy included chemotherapy in 98% of patients, while only 13% underwent radiotherapy, either alone or in combination with chemotherapy.

### Pain severity and metastatic factors

The mean initial Visual Analogue Scale (VAS) pain score was 5 (range: 0-10). TThe severity of pain significantly differed depending on the location of spinal metastases (Table 2). Patients with lumbar spine involvement reported the highest mean pain score (8, range: 5-10), while those with cervical spine metastases had the lowest mean score (4, range: 0-6; P = 0.0003). Patients with multiple metastases also experienced high pain scores, similar to those with isolated lumbar lesions.

**Figure 2:**
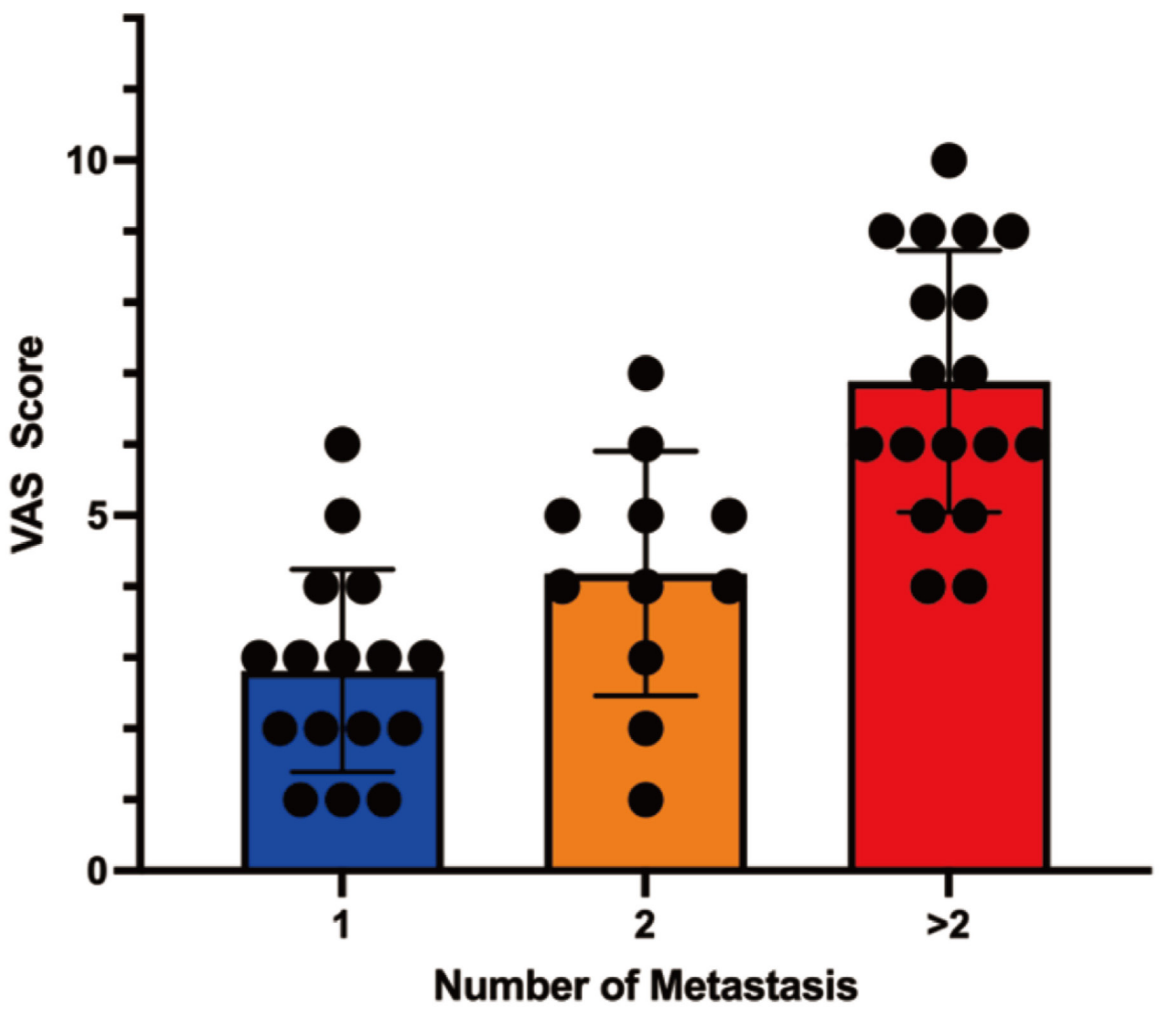
Correlation analysis between Metastasis numbers and pain score.

A strong correlation was obtained between the number of spinal metastases and the severity of pain (P < 0.0001). Patients with a single metastasis had a mean VAS score of 4 (range: 1-6), while those with two metastases reported a mean score of 5 (range: 1-7). Patients with more than two metastases exhibited a mean score of 8 (range: 4-10) (Table 2; Figure 2).

### MRI findings and pain correlations

**Figure 3:**
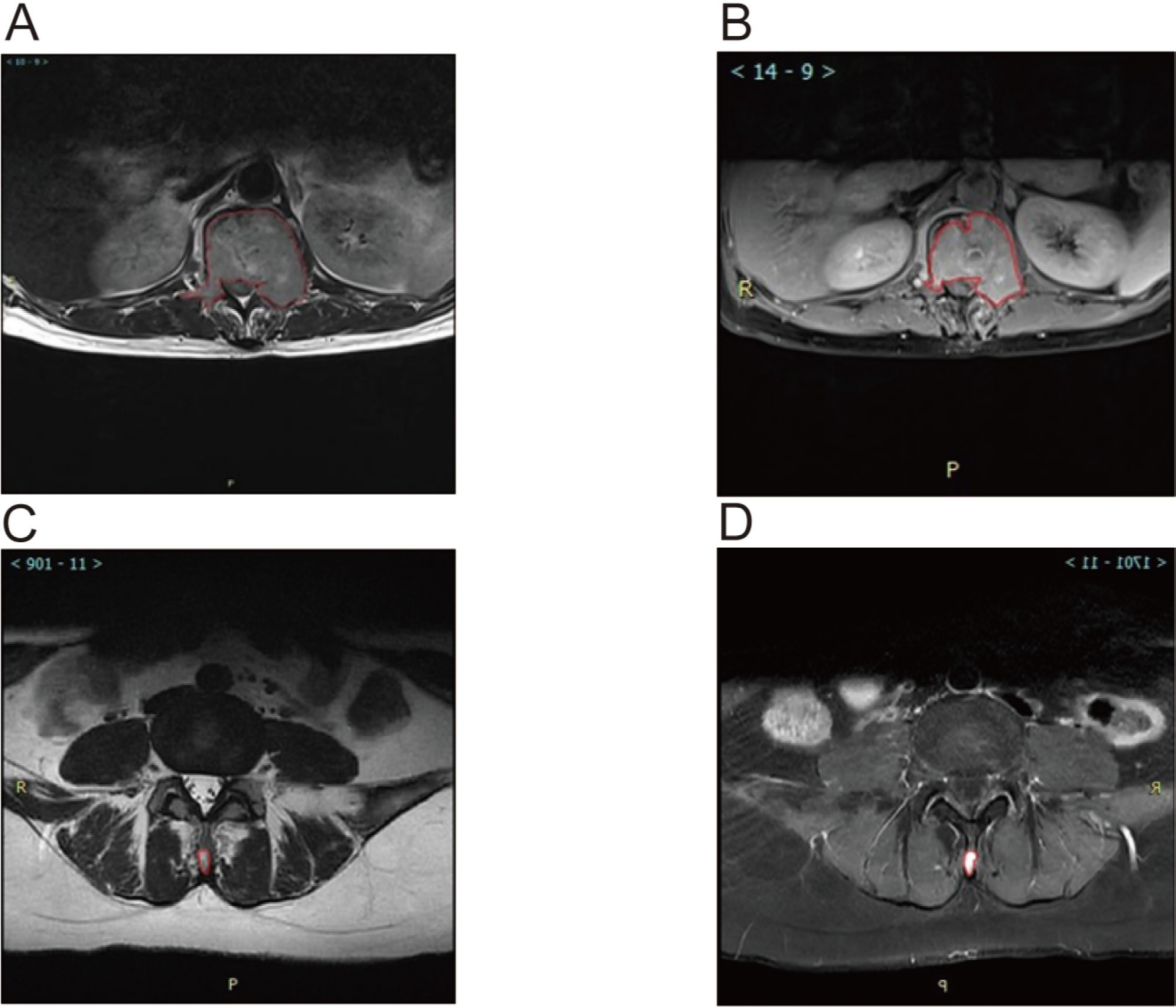
Magnetic Resonance Imaging (MRI) Images showed Metastatic Lesion location and high-intensity zone in Patients With spine metastasis of breast cancer. A. Calulate the proportion of whole cord in the T2 scan image of the lumbar metastasis. B. Calulate the proportion of high indesity area in the T2 scan image of the lumbar metastasis. C. Calulate the proportion of whole cord in the T2 scan image of the Spinous process metastasis. D. Calulate the proportion of high indesity area in the T2 scan image of the Spinous process metastasis.

**Figure 4:**
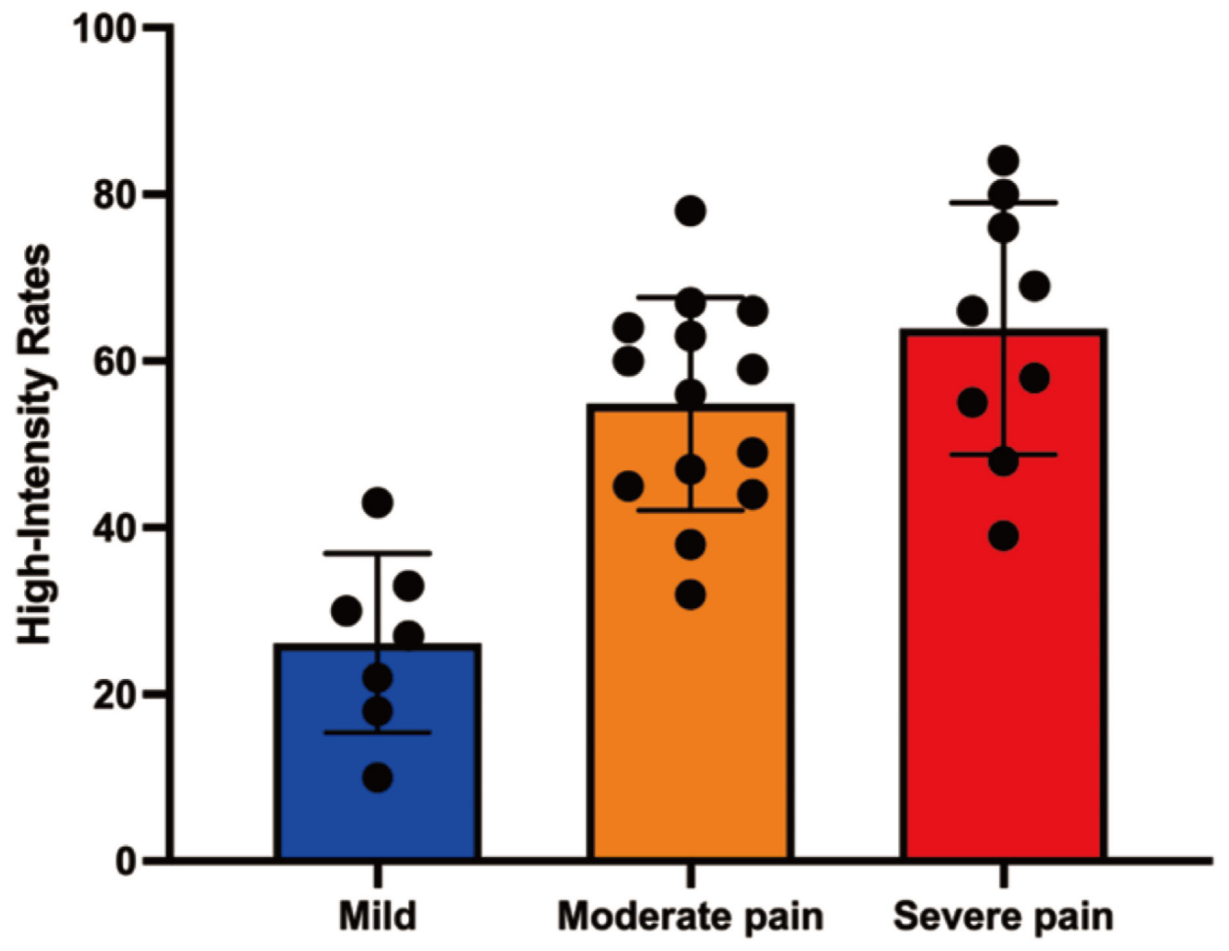
Correlation analysis between high-intensity zone rates and pain score.

MRI analysis revealed significant associations between the ratio of high-intensity zones (RHI) and pain severity (Table 3). The median RHI was 47% (range: 10-84%). Patients with mild pain (VAS 1-3) exhibited a median RHI of 27% (range: 10-43%), whereas those with moderate pain (VAS 4-7) and severe pain (VAS 8-10) had significantly higher median RHIs of 59% (range: 32-78%) and 66% (range: 39-84%), respectively (P < 0.0001). Figure 3 illustrates representative MRI images showing the locations of metastatic lesions and associated high-intensity zones. Correlation analysis confirmed a significant linear relationship between RHI and pain scores (Figure 4).

## Discussion

This study provides evidence that the combination of metastatic sites and MRI-derived features, particularly the ratio of high-intensity zones (RHI), is markedly correlated with the severity of bone pain in breast cancer patients with spinal metastases. These results hold significant practical relevance for elucidating the pathophysiology of bone pain and for optimizing patient management 30-33.

Our results showed that spinal metastases in the lumbar region are associated with significantly higher pain scores compared to those in the cervical or thoracic regions. This may be due to the biomechanical and structural differences in these regions, with the lumbar spine bearing greater mechanical loads, thereby amplifying pain when metastases are present 34, 35. Parallel observations have been made in various forms of neoplastic disease emphasizing the impact of spinal biomechanics on metastatic pain severity 36-38.

The strong correlation between the number of metastatic sites and pain severity further supports the role of tumor burden in exacerbating symptoms 39, 40. Individuals with disseminated disease exhibited markedly elevated discomfort levels relative to those with solitary foci. This aligns with the hypothesis that cumulative skeletal involvement leads to increased mechanical instability, nerve compression, and heightened inflammatory responses 41, 42.

The MRI findings in this study highlight the role of RHI as a potential biomarker for bone pain severity. Higher RHI values may reflect increased tumor activity, bone marrow edema, or inflammatory processes within the metastases, all of which contribute to heightened pain perception. Previous research has suggested that advanced imaging features such as fluorine-18 fluorodeoxyglucose PET can serve as prognostic indicators for pain severity and disease progression in cancer patients^16^. These findings underscore the utility of MRI not only in diagnosing spinal metastases but also in guiding targeted pain management strategies 43, 44.

The integration of imaging data into routine clinical workflows could significantly improve patient outcomes. Identifying patients at high risk of severe pain based on metastatic location, RHI values, and lesion number enables the early initiation of aggressive pain management strategies. For instance, targeted radiotherapy or systemic treatments like bisphosphonates and denosumab may be more effectively applied when guided by these predictive imaging markers 45, 46. Additionally, the ability to stratify patients by pain risk could aid in personalized care planning, potentially improving their overall quality of life 47.

Although this research offers meaningful contributions, certain methodological constraints warrant consideration. The look-back methodology and modest cohort size may constrain the broader applicability of our results 48. Additionally, factors such as individual pain tolerance, psychological status, and concurrent treatments were not accounted for, which may have influenced pain scores 49. Further forward-looking investigations involving expanded populations are required to corroborate these results and further elucidate the mechanisms linking MRI features to pain severity. Exploring the role of advanced imaging modalities, such as functional MRI or positron emission tomography (PET), could provide additional insights into the complex relationship between metastatic burden and pain.

This study provides compelling evidence linking the location of spinal metastases, the extent of high-intensity areas observed on MRI, and the intensity of bone pain experienced by patients with breast cancer 50. Our findings underscore the complex interplay between tumor characteristics and patient-reported symptoms, offering valuable insights for clinical practice.

The observation that lumbar spine metastases are associated with significantly greater pain compared to cervical or thoracic involvement is noteworthy. This disparity likely stems from the unique biomechanical demands placed on the lumbar region, which bears a greater share of the body's weight and is subjected to more movement. The presence of a tumor in this area could exacerbate pain through increased mechanical stress, nerve compression, and local inflammation. This highlights the importance of considering the specific anatomical location of metastases when assessing and managing pain.

Furthermore, the study establishes a robust correlation between the number of metastatic sites and pain severity^51^. Patients with multiple spinal lesions reported markedly higher pain scores compared to those with solitary metastases. This finding reinforces the notion that the overall tumor burden contributes substantially to the intensity of symptoms^52^. The cumulative effect of multiple lesions may result in greater mechanical instability, more widespread nerve irritation, and a more pronounced inflammatory response, collectively leading to increased pain^53^.

The significant correlation between the ratio of high-intensity areas (RHI) on MRI and pain severity adds another layer of complexity to our understanding of the relationship between imaging features and clinical manifestations^54^-^60^. A higher RHI may reflect increased tumor activity, bone marrow edema, or the presence of inflammatory mediators within the metastatic lesion. These factors could all contribute to the heightened pain sensitivity observed in patients with elevated RHI values. These observations underscore the potential of MRI as a tool not only for diagnosing metastases, but also for predicting the severity of associated pain.

The clinical implications of these findings are substantial. By identifying patients at higher risk of experiencing severe pain based on the location, number of metastases, and RHI values, clinicians can proactively implement more aggressive pain management strategies. This may include targeted radiation therapy, systemic treatments such as bisphosphonates or denosumab, or other novel approaches. Furthermore, factors such as individual pain tolerance, psychological status, and concurrent treatments were not accounted for, which may have influenced pain scores. In conclusion, this study demonstrates a significant association between spinal metastatic sites, MRI-derived RHI values, and the severity of bone pain in breast cancer patients. These findings highlight the critical role of MRI not only as a diagnostic tool but also as a predictor of symptomatic outcomes. Incorporating these imaging parameters into clinical practice could enhance symptom management and improve the quality of life for patients with metastatic breast cancer.

## Conclusion

In summary, our study highlights the significant association between spinal metastatic sites, MRI-derived RHI values, and bone pain severity in breast cancer patients. These findings emphasize the critical role of MRI not only as a diagnostic tool but also as a predictor of symptomatic outcomes. Incorporating these imaging parameters into clinical practice could enhance symptom management and improve the quality of life for patients with metastatic breast cancer.

## Figures and Tables

**Figure 1:**
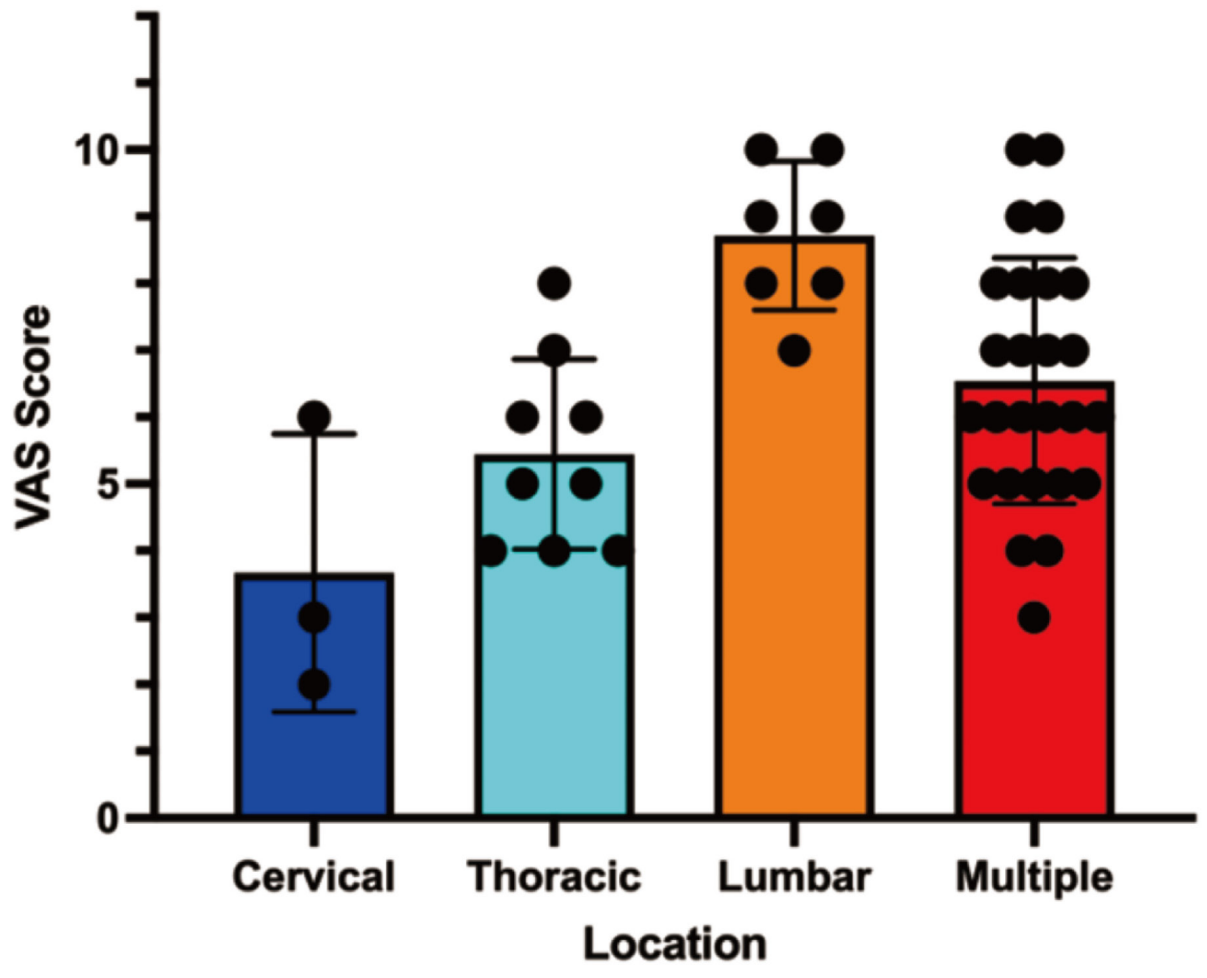
Correlation analysis between Metastasis location and pain score.

**Table 1:** Patient (n= 45) and Metastatic Lesion (n= 45) clinical Characteristics

Characteristic	Number of Patients(% or Range)
Gender	
Male	N=0(0%)
Female	N=45(100%)
Median age (years)	51
Median time of spine metastatic (months)	27
Histologic type	
Luminal A	4(9%)
Luminal B	31(69%)
Her-2 positive	4(9%)
TNBC	6(13%)
Bone metastases (n)	
One	16(36%)
Multiple	29(46%)
Location	
Cervical	3(6%)
Thoracic	9(20%)
Lumbar	7(16%)
Multiple	26(58%)
Pain occurrence	
With pain	39(87%)
Without pain	6(13%)
Time of bone metastasis and pain	
Metachronous	35(90%)
Synchronous	4(10%)
Systematic therapy	
Chemotherapy	44(98%)
Radiotherapy	6(13%)
Both	6(13%)
None	0

**Table 2:** Correlations of Spine Metastasis to Pain Scores

Factor	Mean	Range	P Value
Initial pain score (VSA)	5	0-10	-
Location			0.0003
Cervical	4	0-6	
Thoracic	6	2-8	
Lumbar	8	5-10	
Multiple	8	5-10	
Nunber of Metastasis			<0.0001
1	4	1-6	
2	5	1-7	
over two	8	4-10	

**Table 3:** Correlations of Spine Metastasis high-intensity zone rates to Pain Scores

Factor	Median	Range	P Value
High-intensity zone rates	47	10-84	
Pain serious			<0.0001
Mild pain (1-3)	27	10-43	
Moderate pain (4-7)	59	32-78	
Severe pain (8-10)	66	39-84	
